# F157. HIERARCHICAL PREDICTION ERRORS DURING AUDITORY MISMATCH UNDER PHARMACOLOGICAL MANIPULATIONS: A COMPUTATIONAL SINGLE-TRIAL EEG ANALYSIS

**DOI:** 10.1093/schbul/sby017.688

**Published:** 2018-04-01

**Authors:** Lilian Weber, Andreea Diaconescu, Sara Tomiello, Dario Schöbi, Sandra Iglesias, Christoph Mathys, Helene Haker, Gabor Stefanics, André Schmidt, Michael Kometer, Franz X Vollenweider, Klaas Enno Stephan

**Affiliations:** 1 ETH Zurich; 2 University of Zurich, ETH Zurich; 3 SISSA; 4 University of Basel (UPK); 5 University Hospital of Psychiatry, University of Zurich

## Abstract

**Background:**

A central theme of contemporary neuroscience is the notion that the brain embodies a generative model of its sensory inputs to infer on the underlying environmental causes, and that it uses hierarchical prediction errors (PEs) to continuously update this model. In two pharmacological EEG studies, we investigate trial-wise hierarchical PEs during the auditory mismatch negativity (MMN), an electrophysiological response to unexpected events, which depends on NMDA-receptor mediated plasticity and has repeatedly been shown to be reduced in schizophrenia.

**Methods:**

Study1: Reanalysis of 64 channel EEG data from a previously published MMN study (Schmidt et al., 2012) using a placebo-controlled, within-subject design (N=19) to examine the effect of S-ketamine. Study2: 64 channel EEG data recorded during MMN (between subjects, double-blind, placebo-controlled design, N=73), to examine the effects of amisulpride and biperiden. Using the Hierarchical Gaussian Filter, a Bayesian learning model, we extracted trial-by-trial PE estimates on two hierarchical levels. These served as regressors in a GLM of trial-wise EEG signals at the sensor level.

**Results:**

We find strong correlations of EEG with both PEs in both samples: lower-level PEs show effects early on (Study1: 133ms post-stimulus, Study2: 177ms), higher-level PEs later (Study1: 240ms, Study2: 450ms). The temporal order of these signatures thus mimics the hierarchical relationship of the PEs, as proposed by our computational model, where lower level beliefs need to be updated before learning can ensue on higher levels. Ketamine significantly reduced the representation of the higher-level PE in Study1. (Study2 has not been unblinded.)

**Discussion:**

These studies present first evidence for hierarchical PEs during MMN and demonstrate that single-trial analyses guided by a computational model can distinguish different types (levels) of PEs, which are differentially linked to neuromodulators of demonstrated relevance for schizophrenia. Our analysis approach thus provides better mechanistic interpretability of pharmacological MMN studies, which will hopefully support the development of computational assays for diagnosis and treatment predictions in schizophrenia.

